# National and State Attitudes of US Adults Toward Tobacco-Free School Grounds, 2009–2010

**DOI:** 10.5888/pcd12.150353

**Published:** 2015-12-31

**Authors:** Judy Kruger, Roshni Patel, Michelle C. Kegler, Nancy D. Brener, Brian A. King

**Affiliations:** Author Affiliations: Roshni Patel, DB Consulting Group, Atlanta, Georgia; Michelle C. Kegler, Rollins School of Public Health, Emory University, Atlanta, Georgia; Nancy D. Brener, Division of Adolescent and School Health, National Center for HIV/AIDS, Viral Hepatitis, STD, and TB Prevention, Centers for Disease Control and Prevention, Atlanta, Georgia; Brian A. King, Office on Smoking and Health, National Center for Chronic Disease Prevention and Health Promotion, Centers for Disease Control and Prevention, Atlanta, Georgia.

## Abstract

**Introduction:**

Schools are an important environment for addressing tobacco use among youth. Tobacco-free school policies can help reduce the social acceptability of tobacco use and prevent tobacco initiation among youth. This study assessed attitudes toward tobacco-free school grounds among US adults.

**Methods:**

Data came from the 2009–2010 National Adult Tobacco Survey, a telephone survey of adults aged 18 or older in the 50 US states and District of Columbia. Respondents were considered to have a favorable attitude toward tobacco-free school grounds if they reported tobacco use should be completely banned on school grounds, including fields and parking lots, and at all school events. Data were assessed using descriptive statistics and multivariable logistic regression, overall and by tobacco use status. Correlates were sex, age, race/ethnicity, education, marital status, income, sexual orientation, US region, and whether respondent lived with any children aged 17 years or younger.

**Results:**

Nationally, 86.1% of adults had a favorable attitude toward tobacco-free school grounds, with larger percentages among nontobacco users (91.9%) than current users (76.1%). State prevalence ranged from 80.0% (Kentucky) to 90.9% (Washington). Overall odds of favorable attitudes were higher among nontobacco users (referent, current users), women (referent, men), and adults aged 25 or older (referent, aged 18–24); odds were lower among residents of the South (referent, West) and lesbian, gay, bisexual, or transgender adults (referent, heterosexual or straight).

**Conclusion:**

Nearly 9 in 10 US adults have a favorable attitude toward tobacco-free school grounds, but attitudes vary across states and subpopulations. Opportunities exist to educate the public about the benefits of tobacco-free school grounds, which might help reduce tobacco use among youth.

## Introduction

Tobacco use is the leading cause of preventable disease and death in the United States; cigarette smoking alone is responsible for more than 480,000 deaths and nearly $300 billion in direct medical care and lost productivity each year (1). Approximately 9 in 10 adult smokers first try cigarettes before age 18 (2), and an estimated 21.3% of US adults (3) and 24.6% of US high school students are current tobacco product users (4).

Tobacco-free policies, when fully enforced, are an effective population-level approach to prevent tobacco use initiation and reduce the social acceptability of tobacco use among youth (5–7). Schools are an especially important environment for implementing tobacco-free policies because students spend a considerable amount of time in schools and the area surrounding schools is a common location for youth tobacco use (8,9). Tobacco-free policies can help reduce youth tobacco use initiation and discourage youth from becoming established tobacco users by changing environmental cues and social norms about the acceptability of smoking, reducing the number of places where youth can use or obtain tobacco, and reducing opportunities to view adult role modeling tobacco use (10–12). Other potential benefits of tobacco-free school policies include decreased school maintenance costs due to tobacco litter and reduced fire risk (1), as well as reduced smoking and secondhand smoke exposure among staff, visitors, and students (6,7,10,14).

Public attitudes and changing social norms are an important precursor to the establishment of tobacco-free environments (7) and can play a critical role in facilitating the adoption of tobacco-free policies and in preventing tobacco use initiation among youth (10). To date, no studies have characterized public attitudes toward tobacco-free school grounds as a means for informing efforts to reduce the social acceptability and use of tobacco in this environment. The objective of this study was to 1) assess the national and state prevalence of favorable attitudes toward tobacco-free school grounds among US adults and 2) examine sociodemographic correlates of favorable attitudes toward tobacco-free school grounds, overall and by current tobacco use status. The findings from this research could help inform efforts to educate the public about the benefits of tobacco-free environments and the importance of adopting tobacco-free school policies.

## Methods

### Sample

Data were obtained from the 2009–2010 National Adult Tobacco Survey (NATS), a national landline and cellular telephone survey of noninstitutionalized civilian adults aged 18 or older residing in the 50 US states and the District of Columbia (15). The 2009–2010 NATS used a stratified, multistage probability design to yield data representative at both national and state levels. For the landline component, each state was allocated an equal target sample size (n = 1,863) to ensure adequate precision for state estimates. For the cellular telephone component, each state was allocated a sample size in proportion to its population. Louisiana, New Jersey, and Oklahoma added to their landline and cellular telephone target sample size; Delaware, Georgia, Iowa, North Dakota, Pennsylvania, South Carolina, and Virginia added to the landline target only.

The study design has been described in detail elsewhere (15). In brief, respondent selection varied by telephone type. For landline numbers, one adult was randomly selected from each eligible household. For cellular numbers, the adult reached was selected if a cellular telephone was the only way he or she could be reached by telephone at home. Interviews were administered from October 20, 2009, to February 28, 2010, and were conducted in English and Spanish. In total, 118,581 interviews were conducted (landline, 110,634; cellular, 7,947). The overall Council of American Survey and Research Organizations (CASRO) response rate, defined as the number of completed interviews divided by the number of eligible respondents in the sample, was 37.6% (landline, 40.4%; cellular, 24.9%) (16). The overall cooperation rate, defined as the number of completed interviews divided by the number of eligible respondents who were successfully reached by an interviewer, was 62.3% (landline, 61.9%; cellular, 68.7%). State CASRO response rates ranged from 28.2% in New Jersey to 49.3% in Vermont, and cooperation rates ranged from 52.9% in Louisiana to 72.4% in Vermont.

### Measures

#### Attitudes toward tobacco-free school grounds

Attitudes toward tobacco-free school grounds were assessed by the question, “Should tobacco use be completely banned on school grounds, including fields and parking lots, and at all school events even for teachers and other adults?” Response options were yes or no. Respondents who selected yes were classified as having a favorable attitude toward tobacco-free school grounds.

Respondents were categorized as being a current tobacco user or being a nontobacco user. Current tobacco users were classified according to answers to questions on the use of 6 tobacco products: cigarettes, cigars/cigarillos/filtered little cigars, pipes, water pipes, chewing tobacco/snuff/dip, or snus. Current tobacco users were defined as those who reported smoking 100 cigarettes or more during their lifetime and now smoking every day or some days and/or using cigars/cigarillos/filtered little cigars, pipes, water pipes, chewing tobacco/snuff/dip, or snus on at least one day in the previous 30 days. Nontobacco users were defined as those not currently using any of the 6 tobacco products.

The following sociodemographic characteristics were assessed: sex (male or female), age (18–24, 25–44, 45–64, or ≥65 y), race/ethnicity (non-Hispanic white, non-Hispanic black, non-Hispanic Asian, non-Hispanic American Indian/Alaska Native, non-Hispanic Native Hawaiian/Pacific Islander, non-Hispanic multirace, non-Hispanic other, or Hispanic), education (0–12 years [no diploma], general educational development (GED), high school graduate, some college [no degree], associate degree, undergraduate degree, or graduate degree), marital status (single/separated/divorced/widowed or married/living with a partner), annual household income (<$20,000, $20,000–$49,999, $50,000–$99,999, ≥$100,000, or not specified), US Census region (West, Northeast, Midwest, or South), sexual orientation (heterosexual or straight; lesbian, gay, bisexual, or transgender [LGBT], or not specified), and whether the respondent lived with any children aged 17 years or younger (yes or no). Because of small sample sizes, those who self-identified as lesbian, gay, bisexual, or transgender were combined into a single category, LGBT. 

### Analysis

Data were analyzed using SAS-Callable SUDAAN 10 (RTI International) and weighted to adjust for the differential probabilities of selection and response. For states with a small number of cellular respondents, the use of both landline and cellular telephone data resulted in a large unequal weighting effect and, therefore, large estimated variances of survey estimates and small effective sample sizes. As a result, the national and state estimates were calculated differently. For national estimates, both cellular telephone and landline respondents were included. For state estimates, cellular telephone respondents were included only for the 12 states that had a cellular sample of 200 or more respondents (California, Florida, Georgia, Illinois, Louisiana, New Jersey, New York, North Carolina, Ohio, Oklahoma, Pennsylvania, and Texas).

Descriptive analyses, including point estimates and 95% confidence intervals (CIs), were conducted to assess overall attitudes toward tobacco-free school grounds at the national and state levels. National estimates also were assessed by sociodemographic characteristics. Global χ^2^ tests were used to determine significant differences between groups (*P* < .05). The relative standard error for all presented estimates was less than 30%. Additionally, multivariate logistic regression models were conducted to assess favorable attitudes toward tobacco-free school grounds among 3 groups: all adults, current tobacco users only, and nontobacco users only.

## Results

### National attitudes

#### Overall

Nationally, 86.1% of US adults reported favorable attitudes toward tobacco-free school grounds (Table 1). The prevalence of favorable attitudes toward tobacco-free school grounds differed by sex, age, education, and presence of children aged 17 years or younger in the household.

**Table 1:** Prevalence and Adjusted Odds Ratio of Favorable Attitudes Toward Tobacco-Free School Grounds^a^, by Selected Characteristics, National Adult Tobacco Survey, 2009–2010

Characteristic	Overall		Current Tobacco User^b^		Nontobacco User	
	% (95% CI) (n = 68,489)	AOR (95% CI)	% (95% CI) (n = 18,802)	AOR (95% CI)	% (95% CI) (n = 49,687)	AOR (95% CI)
**Overall**	86.1 (85.5–86.7)	---	76.1 (74.9–77.2)	1 [Ref]^c^	91.9 (91.3–92.5)	3.0 (2.7–3.4)^d^
**Sex**						
Male	80.9 (79.8–82.0)	1 [Ref]	74.4 (72.8–75.9)	1 [Ref]	88.5 (87.0–90.1)	1 [Ref]
Female	90.0 (89.3–90.6) **e**	1.5 (1.3–1.6)^d^	78.9 (77.1–80.6) **e**	1.3 (1.1–1.4)^d^	93.5 (92.9–94.0) **e**	1.7 (1.4–2.0)^d^
**Age, y**						
18–24	79.1(77.1–81.0)	1 [Ref]	67.5 (64.2–70.9)	1 [Ref]	88.4 (86.2–90.6)	1 [Ref]
25–44	86.1 (85.0–87.2)	1.4 (1.2–1.6)^d^	78.3 (76.4–80.1)	1.6 (1.4–2.0)^d^	91.5 (90.2–92.8)	1.0 (0.7–1.4)
45–64	87.1 (86.3–88.0)	1.5 (1.3–1.7)^d^	77.1 (75.3–78.9)	1.6 (1.3–1.9)^d^	92.7 (91.9–93.5)	1.2 (0.9–1.6)
≥65	91.5 (90.7–92.4) **e**	1.9 (1.6–2.3)^d^	80.7 (77.8–83.6) **e**	1.9 (1.5–2.5)^d^	93.9 (93.1–94.8) **e**	1.7 (1.2–2.2)^d^
**Race/ethnicity**						
Non-Hispanic white	86.0 (85.4–86.6)	1 [Ref]	75.2 (73.9–76.5)	1 [Ref]	92.9 (92.4–93.4)	1 [Ref]
Non-Hispanic black	86.1 (84.4–87.9)	1.0 (0.9–1.2)	79.3 (75.6–83.0)	1.3 (1.0–1.7)	89.5 (87.8–91.3)	0.8 (0.6–1.0)
Non-Hispanic Asian	87.2 (80.2–94.1)	0.8 (0.4–1.6)	78.0 (66.7–89.3)	1.2 (0.6–2.4)	88.9 (80.8–96.9)	0.7 (0.3–1.5)
Non-Hispanic AI/AN	81.9 (77.4–86.3)	1.0 (0.7–1.4)	75.6 (69.1–82.2)	1.0 (0.7–1.5)	92.2 (88.1–96.3)	1.0 (0.5–1.7)
Non-Hispanic NH/PI	82.1 (73.2–90.9)	0.8 (0.4–1.3)	60.1 (42.5–77.7)	0.5 (0.2–1.0)	95.4 (91.5–99.2)	1.8 (0.7–4.5)
Non-Hispanic multirace	80.7 (74.6–86.8)	0.8 (0.5–1.2)	71.7 (63.8–79.6)	0.8 (0.6–1.2)	89.1 (79.3–98.9)	0.7 (0.3–1.8)
Non-Hispanic other	75.6 (63.1–88.2)	0.6 (0.3–1.2)	61.2 (41.0–81.4)	0.5 (0.2–1.3)	90.6 (81.8–99.3)	0.7 (0.3–2.2)
Hispanic	87.7 (85.6–89.8)	1.1 (0.9–1.4)	80.3 (75.5–85.0)	1.3(1.0–1.8)	90.9 (88.8–93.0)	1.0 (0.7–1.3)
**Education**						
0–12 years (no diploma)	85.8 (83.9–87.7)	1 [Ref]	80.5 (77.5–83.6)	1 [Ref]	90.3 (88.1–92.5)	1 [Ref]
GED	81.9 (78.2–85.6)	1.0 (0.7–1.3)	77.5 (72.4–82.5)	0.9 (0.6–1.3)	90.9 (86.4–95.4)	1.0 (0.6–1.8)
High school graduate	84.2 (83.0–85.5)	0.8 (0.7–1.0)	73.7 (71.5–75.8)	0.7 (0.6–0.9)^d^	91.1 (89.7–92.6)	1.0 (0.8–1.4)
Some college (no diploma)	85.5 (84.2–86.8)	0.8 (0.7–1.0)	74.0 (71.3–76.7)	0.7 (0.6–0.9)^d^	92.1 (90.9–93.4)	1.1 (0.8–1.5)
Associate degree	87.1 (85.8–88.3)	0.9 (0.7–1.1)	76.7 (73.9–79.5)	0.8 (0.0–1.0)	92.9 (91.7–94.0)	1.2 (0.8–1.6)
Undergraduate degree	88.3 (87.2–89.4)	0.9 (0.7–1.1)	75.3 (72.4–78.3)	0.8 (0.6–1.0)	92.3 (91.2–93.4)	1.1 (0.8–1.5)
Graduate degree	91.3 (90.1–92.4) **e**	1.0 (0.8–1.3)	75.7 (71.3–80.0) **e**	0.7 (0.5–1.0)	94.4 (93.3–95.4) **e**	1.4 (1.0–2.0)
**Marital status**						
Single, separated, divorced, or widowed	83.9 (82.9–84.9)	1 [Ref]	77.9 (76.4–79.5)	1 [Ref]	92.9 (92.2–93.5)	1 [Ref]
Married or partnered	87.9 (87.2–88.6)	1.1 (1.0–1.3)	74.2 (72.4–76.0)	1.1 (1.0–1.3)	90.6 (89.5–91.8)	1.1 (0.9–1.3)
**Household income, $**						
<20,000	84.0 (82.3–85.8)	1 [Ref]	76.8 (73.8–79.8)	1 [Ref]	90.5 (88.7–92.3)	1 [Ref]
20,000–49,999	85.8 (84.7–86.8)	1.1 (0.9–1.3)	76.9 (75.0–78.8)	1.1 (0.9–1.4)	91.6 (90.3–92.8)	1.1 (0.9–1.5)
50,000–99,999	87.3 (86.4–88.2)	1.2 (1.0–1.4)	75.7 (73.6–77.8)	1.1 (0.9–1.3)	92.9 (92.1–93.7)	1.3 (1.0–1.7)
≥100,000	87.7 (86.4–89.1)	1.2 (0.9–1.4)	73.8 (70.3–77.4)	1.0 (0.8–1.3)	93.4 (92.3–94.5)	1.4 (1.0–1.8)
Not specified	83.4 (80.2–86.5)	0.9 (0.7–1.2)	73.0 (66.7–79.3)	1.0 (0.7–1.4)	88.0 (84.4–91.5)	0.8 (0.6–1.3)
**US Census region** f						
West	87.9 (86.3–89.5)	1 [Ref]	78.8 (75.7– 81.9)	1 [Ref]	92.6 (90.8–94.3)	1 [Ref]
Northeast	87.7 (86.5–88.9)	0.9 (0.8–1.1)	78.5 (76.0–80.9)	1.0 (0.8–1.2)	92.0 (90.7–93.3)	0.9 (0.7–1.2)
Midwest	85.3 (84.1–86.4)	0.8 (0.7–1.0)	75.1 (72.8–77.4)	0.8 (0.6–1.0)	91.9 (90.8–93.0)	0.9 (0.7–1.1)
South	84.8 (83.9–85.7)	0.8 (0.7–0.9)^d^	74.3 (72.4–76.1)	0.7 (0.6–0.9)^d^	91.5 (90.6–92.4)	0.9 (0.7–1.1)
**Sexual orientation**						
Heterosexual or straight	86.4 (85.8–87.0)	1 [Ref]	76.1 (74.9–77.4)	1 [Ref]	92.2 (91.6–92.9)	1 [Ref]
LGBT	77.4 (73.1–81.7)	0.7 (0.5–0.9)^d^	71.5 (65.4–77.6)	0.8 (0.6–1.1)	83.9 (77.7–90.0)	0.5 (0.3–0.9)^d^
Not specified	87.4 (82.8–92.0)	0.8 (0.5–1.3)	92.9 (87.8–97.9)	3.5 (1.6–7.7)^d^	86.3 (80.9–91.7)	0.6 (0.4–1.1)
**Children aged** ≤**17 y living in household**						
No	85.5 (84.7–86.2)	1 [Ref]	77.8 (75.9–79.8)	1 [Ref]	92.3 (91.4–93.2)	1 [Ref]
Yes	87.0 (86.1–87.9) **e**	1.2 (1.0–1.3)	74.7 (73.2–76.2) **e**	1.1 (1.0–1.3)	91.6 (90.8–92.5) **e**	1.2 (1.0–1.5)

Abbreviations: — , does not apply; AI/AN, American Indian/Alaska Native; AOR, adjusted odds ratio; CI, confidence interval; GED, general educational development; LGBT, lesbian, gay, bisexual, or transgender; NH/PI, Native Hawaiian/Pacific Islander; Ref, reference.

a Favorable attitudes toward tobacco-free school grounds was defined as a response of yes to the question “Should tobacco use be completely banned on school grounds, including fields and parking lots, and at all school events even for teachers and other adults?”

b Current tobacco users were defined as those who reported smoking ≥100 cigarettes during their lifetime and now smoking every day or some days and/or using cigars/cigarillos/filtered little cigars, pipes, water pipes, chewing tobacco/snuff/dip, or snus on ≥1 day in the previous 30 days.

c Current tobacco user is the reference group for the overall comparison between nontobacco user and current tobacco user.

d Significantly different from referent group; determined by multivariable logistic regression (*P* < .05). Odds ratios adjusted for all covariates listed in table.

e Significantly different from referent group in same column; determined by χ^2^ test (*P* < .05). Referent group for each category was as follows: sex, male; age, 18–24 y; race/ethnicity, non-Hispanic white; education, 0–12 y (no diploma); marital status, single, separated, divorced, or widowed; household income, <$20,000; US region, west; sexual orientation, heterosexual or straight; no children aged ≤17 y living in household; and current tobacco user.

f Northeast: Connecticut, Maine, Massachusetts, New Jersey, New Hampshire, New York, Pennsylvania, Rhode Island, and Vermont. Midwest: Illinois, Indiana, Iowa, Kansas, Michigan, Minnesota, Missouri, Nebraska, North Dakota, Ohio, South Dakota, and Wisconsin. South: Alabama, Arkansas, Delaware, District of Columbia, Florida, Georgia, Kentucky, Louisiana, Maryland, Mississippi, North Carolina, Oklahoma, South Carolina, Tennessee, Texas, Virginia and West Virginia. West: Alaska, Arizona, California, Colorado, Hawaii, Idaho, Montana, Nevada, New Mexico, Oregon, Utah, Washington, and Wyoming.

In multivariable analyses, odds of favorable attitudes toward tobacco-free school grounds were higher among nontobacco users (odds ratio [OR], 3.0; 95% CI, 2.7–3.4) than among current tobacco users. Odds of favorable attitudes were higher among women (OR, 1.5; 95% CI, 1.3–1.6) than among men and among adults aged 25 to 44 (OR, 1.4; 95% CI, 1.2–1.6), aged 45 to 64 (OR, 1.5; 95% CI, 1.3–1.7), and aged 65 or older (OR, 1.9; 95% CI, 1.6–2.3) than among adults aged 18 to 24. Odds were lower among those living in the South (OR, 0.8; 95% CI, 0.7–0.9) than among those living in the West and among LBGT adults (OR, 0.7; 95% CI, 0.5–0.9) than among heterosexual or straight adults.

#### Current tobacco users

Nationally, 76.1% of current tobacco users reported favorable attitudes toward tobacco-free school grounds (Table 1). Prevalence estimates differed by sex, age, education, and presence of children aged 17 years or younger in the household.

Odds of favorable attitudes toward tobacco-free school grounds among current tobacco users were higher among women (OR, 1.3; 95% CI, 1.1–1.4) than among men (Table 1); adults aged 25 to 44 (OR, 1.6; 95% CI, 1.4–2.0), aged 45 to 64 (OR, 1.6; 95% CI, 1.3–1.9), and aged 65 or older (OR, 1.9; 95% CI, 1.5–2.5) than among those aged 18 to 24; and among those who did not specify sexual orientation (OR, 3.5; 95% CI, 1.6–7.7) than among heterosexual or straight adults. Odds were lower among high school graduates (OR, 0.7; 95% CI, 0.6–0.9) and those with some college education (OR, 0.7; 95% CI, 0.6–0.9) than among those with 0 to 12 years of education and no diploma and among adults living in the South (OR, 0.7; 95% CI, 0.6–0.9) than among adults living in the West.

#### Nontobacco users

Nationally, 91.9% of nontobacco users reported favorable attitudes toward tobacco-free school grounds (Table 1). Prevalence estimates differed by sex, age, education, and presence of children aged 17 years or younger in the household.

Odds of favorable attitudes toward tobacco-free school grounds among nontobacco users were higher among women (OR, 1.7; 95% CI, 1.4–2.0) than among men and among adults aged 65 or older (OR, 1.7; 95% CI, 1.2–2.2) than among those aged 18 to 24. Odds of favorable attitudes toward tobacco-free school grounds were lower among LGBT adults (OR, 0.5; 95% CI, 0.3–0.9) than among heterosexual or straight adults.

### State-specific attitudes

By state, favorable attitudes toward tobacco-free school grounds ranged from 80.0% in Kentucky to 90.9% in Washington (Table 2). Among current tobacco users, prevalence of favorable attitudes toward tobacco-free school grounds ranged from 67.0% in Utah to 85.2% in Washington. Among nontobacco users, prevalence ranged from 87.5% in Nevada to 96.5% in South Dakota. Oregon was the only state in which no significant difference was observed between current tobacco users and nontobacco users.

**Table 2:** National and State Prevalence of Favorable Attitudes Toward Tobacco-Free School Grounds^a^, by Tobacco Use Status, National Adult Tobacco Survey, 2009–2010

State	N	Overall (n = 59,365)	Current Tobacco User^b^ (n = 13,746)	Nontobacco User (n = 45,619)
Alabama	1,022	83.9 (80.3–87.5)	73.2 (66.1–80.2)	91.5 (88.1–94.9)
Alaska	871	86.8 (83.2–90.5)	82.0 (75.8–88.2)	91.0 (86.8–95.2)
Arizona	854	87.5 (83.1–91.8)	76.7 (66.3–87.1)	91.9 (87.9–95.8)
Arkansas	1,536	85.2 (82.1–88.4)	78.6 (73.6–83.6)	90.4 (86.3–94.5)
California^c^	1,261	88.4 (85.5–91.2)	78.7 (72.6–84.8)	92.5 (89.4–95.6)
Colorado	873	83.7 (79.1–88.2)	73.8 (64.2–83.4)	89.5 (85.2–93.9)
Connecticut	887	85.5 (80.8–90.2)	76.2 (65.4–87.0)	89.4 (84.5–94.2)
Delaware	1,008	85.5 (81.8–89.1)	75.1 (67.8–82.4)	91.2 (87.4–95.1)
Washington, DC	740	86.6 (82.0–91.2)	79.7 (70.1–89.3)	90.7 (85.9–95.5)
Florida^c^	1,133	86.4 (83.8–89.0)	76.0 (70.3–81.6)	91.9 (89.3–94.6)
Georgia^c^	2,644	84.5 (82.0–87.0)	76.2 (71.1–81.3)	89.1 (86.4–91.8)
Hawaii	919	90.4 (87.5–93.2)	81.0 (73.7–88.3)	94.9 (92.8–97.0)
Idaho	949	90.0 (87.0–93.0)	80.2 (72.1–88.3)	93.8 (91.4–96.2)
Illinois^c^	1,010	84.9 (81.8–88.1)	75.2 (68.9–81.6)	90.4 (87.0–93.7)
Indiana	983	85.3 (81.7–88.8)	73.0 (65.7–80.2)	93.8 (91.1–96.5)
Iowa	1,091	90.1 (87.2–93.1)	79.2 (72.8–85.6)	95.3 (92.3–98.4)
Kansas	980	85.7 (82.4–89.1)	76.1 (68.8–83.4)	90.5 (87.1–93.9)
Kentucky	936	80.0 (75.9–84.0)	70.6 (63.6–77.6)	89.6 (85.9–93.3)
Louisiana^c^	3,463	83.1 (80.9–85.4)	72.8 (68.5–77.2)	90.6 (88.4–92.7)
Maine	1,018	90.7 (88.1–93.2)	83.7 (78.3–89.2)	94.6 (92.1–97.1)
Maryland	900	88.6 (84.9–92.3)	75.6 (65.2–85.9)	93.4 (90.5–96.3)
Massachusetts	898	87.3 (83.3–91.3)	74.2 (63.2–85.1)	93.2 (90.9–95.5)
Michigan	913	86.4 (82.7–90.1)	73.3 (64.9–81.7)	94.6 (92.5–96.7)
Minnesota	846	90.0 (86.9–93.1)	78.3 (70.6–86.0)	95.8 (93.7–98.0)
Mississippi	1,002	89.3 (86.3–92.4)	82.4 (76.2–88.5)	94.4 (91.9–96.9)
Missouri	982	83.8 (79.5–88.1)	71.8 (62.9–80.7)	91.9 (88.9–94.8)
Montana	898	87.5 (83.9–91.1)	78.5 (70.9–86.2)	94.0 (91.5–96.5)
Nebraska	988	85.3 (81.5–89.0)	72.8 (64.6–81.0)	91.3 (87.7–95.0)
Nevada	878	81.9 (77.8–86.1)	74.1 (66.7–81.5)	87.5 (82.9–92.1)
New Hampshire	974	90.3 (87.6–93.0)	79.7 (72.8–86.7)	95.7 (94.1–97.3)
New Jersey^c^	2,055	87.5 (85.5–89.5)	77.9 (73.3–82.5)	91.5 (89.4–93.5)
New Mexico	842	87.7 (84.0–91.4)	77.6 (69.5–85.7)	93.6 (90.1–97.0)
New York^c^	1,153	89.1 (86.6–91.6)	84.4 (79.7–89.0)	91.1 (88.1–94.2)
North Carolina^c^	1,023	81.9 (78.6–85.3)	69.5 (63.1–75.9)	90.8 (87.6–93.9)
North Dakota	1,156	87.9 (84.1–91.6)	77.0 (69.1–84.9)	93.6 (89.8–97.3)
Ohio^c^	1,081	82.3 (79.6–85.0)	74.2 (69.2–79.3)	88.1 (85.2–91.0)
Oklahoma^c^	1,965	85.6 (83.6–87.5)	76.1 (72.4–79.7)	94.5 (93.0–95.9)
Oregon^d^	912	89.0 (85.1–92.9)	84.6 (78.8–90.4)	91.5 (86.1–96.9)
Pennsylvania^c^	1,803	87.1 (85.1–89.2)	75.6 (71.0–80.2)	93.8 (92.1–95.5)
Rhode Island	1,005	86.0 (82.3–89.7)	80.0 (73.2–86.8)	89.4 (85.0–93.8)
South Carolina	2,643	85.8 (83.5–88.1)	77.4 (72.9–82.0)	90.8 (88.3–93.2)
South Dakota	1,034	89.8 (87.1–92.5)	77.9 (71.2–84.5)	96.5 (95.0–98.0)
Tennessee	957	85.2 (81.9–88.5)	74.4 (67.5–81.2)	93.0 (90.3–95.7)
Texas^c^	1,206	85.9 (83.4–88.5)	76.0 (70.7–81.3)	91.4 (88.9–94.0)
Utah	1,282	90.0 (86.8–93.1)	67.0 (54.7–79.4)	95.2 (93.1–97.3)
Vermont	983	89.5 (86.0–92.9)	78.6 (70.7–86.5)	95.8 (93.6–98.0)
Virginia	1,129	84.3 (81.0–87.6)	74.9 (68.7–81.2)	89.3 (85.5–93.1)
Washington	909	90.9 (88.5–93.2)	85.2 (80.0–90.4)	93.9 (91.4–96.3)
West Virginia	1,005	87.5 (84.4–90.6)	77.7 (71.5–84.0)	95.1 (93.1–97.0)
Wisconsin	890	90.4 (86.8–93.9)	80.9 (72.5–89.2)	95.1 (91.9–98.2)
Wyoming	875	83.5 (79.0–87.9)	70.8 (62.1–79.4)	93.9 (91.5–96.3)
United States	59,365	86.4 (85.8–87.0)	76.4 (75.2–77.6)	91.9 (91.3–92.6)

Abbreviation: CI, confidence interval.

a Favorable attitudes toward tobacco-free school grounds was defined as a response of yes to the question, “Should tobacco use be completely banned on school grounds, including fields and parking lots, and at all school events even for teachers and other adults?”

b Current tobacco users were defined as those who reported smoking ≥100 cigarettes during their lifetime and now smoking every day or some days and/or using cigars/cigarillos/filtered little cigars, pipes, water pipes, chewing tobacco/snuff/dip, or snus on ≥1 day in the previous 30 days.

c Calculated among landline and cellular telephone respondents. All other estimates calculated among landline respondents only.

d Difference between current tobacco user and nontobacco user was significant for all states except Oregon; determined by χ^2^ test (*P* < .05).

## Discussion

This study is the first to examine public attitudes toward tobacco-free school grounds among national and state representative samples of US adults. The findings indicate that most US adults have favorable attitudes toward tobacco-free school grounds, including 9 in 10 nontobacco users and nearly 8 in 10 current tobacco users. Reasons for the high level of favorable attitudes toward tobacco-free schools may be related to shifting social norms on the acceptability of smoking and tobacco use (17,18). To prevent initiation and reduce tobacco use among youth, efforts are warranted to develop and enforce tobacco-free school policies that prohibit all forms of tobacco use on school grounds or campuses, in all school buses or other vehicles used to transport students, and at school functions away from school property (19). Tobacco-free school policies are critical to promote tobacco-free norms and protect nonsmokers, particularly youth, from secondhand smoke exposure. These policies, in conjunction with the adoption of proven tobacco control interventions, such as tobacco price increases, hard-hitting media campaigns, and comprehensive smoke-free policies in indoor public places (8,13), can reduce smoking among adults and youth (20).

The recent diversification of the tobacco landscape with new and emerging products underscores the importance of implementing tobacco-free policies and expanding existing smoke-free policies to include all forms of tobacco use on school grounds. For example, the number of high school students who are using nonconventional tobacco products, such as electronic cigarettes (e-cigarettes), increased during 2011–2014 (4). Tobacco use among youth in any form — whether combustible, noncombustible, or electronic — is unsafe, and prohibiting the use of all tobacco products on school grounds by students, school staff, parents, and visitors, can help reduce youth tobacco use and initiation (1,2).

However, despite the changing landscape of tobacco products and increased use of new and emerging products among youth, progress toward implementing tobacco-free school policies has been limited. The US Department of Health and Human Services established a *Healthy People 2020 *objective (Tobacco use [TU]-15) to increase tobacco-free environments in schools, including all school facilities, property, vehicles, and school events (21). In addition, the Centers for Disease Control and Prevention included tobacco-free schools in guidelines for school health programs to prevent tobacco use and addiction (22). However, the percentage of middle schools (58.7%) and high schools (66.1%) with such policies is well below the 100% goal of *Healthy People 2020 *(23). Moreover, although the 1994 Pro-Children Act required all federally funded schools to prohibit tobacco smoking in all indoor settings (24), states and localities vary on whether smoking is allowed in outdoor settings and whether other forms of tobacco use (eg, smokeless tobacco) are prohibited in indoor and outdoor settings. Currently, 19 states have laws that prohibit smoking on the campuses of private and public kindergarten–grade 12 schools (23).

Given the high level of favorable attitudes toward tobacco-free school grounds found in this study, coupled with the limited progress in expanding coverage of such policies, efforts are warranted to expand state and local tobacco-free policies to include all forms of tobacco use, both indoors and outdoors, on school grounds and at off-campus school functions. The implementation of such policies could be facilitated by assistance from state health departments, many of which have developed model policies and provide technical assistance to school districts seeking to adopt and implement tobacco-free policies (25). However, it is critical that such efforts at schools are implemented in coordination with additional population-based interventions to reduce the social acceptability of tobacco use among youth (2). These interventions include increasing the price of tobacco products, executing comprehensive smoke-free policies in indoor areas of worksites and public places, limiting youth access to tobacco products, and implementing hard-hitting anti-tobacco mass media campaigns (25,26).

This study found differences among states in favorable attitudes toward tobacco-free school grounds. Although it was not possible to rigorously assess the relationship between favorable attitudes and other tobacco-related factors with the present data set, favorable attitudes were generally lower in states that had a higher prevalence of smoking and used fewer evidence-based interventions, such as population-based strategies that set a higher minimum price for cigarettes or tobacco products and comprehensive smoke-free laws (23,25). For example, overall favorable attitudes toward for tobacco-free school grounds were highest in states such as Utah and Hawaii and lowest in Kentucky. Previous research noted similar state variation in the adoption of tobacco-free school policies. In 2012, the percentage of schools that prohibited the use of all tobacco (cigarettes, smokeless tobacco, cigars, and pipes) by all occupants (students, faculty and school staff, and visitors) in all areas (school buildings, outside on school grounds, on school buses or other vehicles used to transport students, and at off-campus, school-sponsored events) during all times (school hours and nonschool hours) ranged from 32.5% (South Dakota) to 80.4% (West Virginia) (27). Thus, more targeted efforts may be warranted to increase the adoption of tobacco-free school policies in certain states, particularly those with the greatest burden of tobacco use and least protection by proven population-based interventions.

Differences in favorable attitudes toward tobacco-free school grounds were also found by tobacco use status and other sociodemographic characteristics. Current tobacco users were generally less likely to have a favorable attitude than nontobacco users. Additionally, women and older adults were more likely to have favorable attitudes, which is consistent with research showing these groups are more supportive of tobacco-free environments (28) and interventions to reduce youth initiation (eg, minors’ access laws, funding for youth tobacco prevention programs) (29). Differences by educational level are probably related to differences in the receptivity of tobacco-related health messages and understanding about the dangers of tobacco use (1,2). Additionally, among nontobacco users, a lower proportion of LGBT individuals reported favorable attitudes toward tobacco-free school grounds than their heterosexual or straight counterparts. These differences may be due to variations in harm perceptions and the influence of attitudes about secondhand smoke exposure in select populations (30) and suggests that formative research may be needed to develop educational messages that reach and resonate with certain subpopulations to expand support for tobacco-free policies on school grounds (7,20).

This study has several limitations. First, data were self-reported and therefore are subject to recall and response bias. Second, it was not possible to determine estimates for each category of current tobacco use (eg, cigarettes, cigars/cigarillos/filtered little cigars, pipes, water pipes, chewing tobacco/snuff/dip, or snus) because of the small sample size and the large number of estimates with high relative standard errors for certain subpopulations. Third, to prevent large variances of survey estimates and small effect sample sizes, cellular telephone respondents were included only for the 12 states with more than 200 cellular telephone respondents. However, cellular respondents were included in all national estimates and in state estimates for the 12 states that had a sufficient sample size. Fourth, the survey had a response rate of 37.6%; low response rates can increase the potential for bias. Finally, data were collected in 2009–2010; because of shifts in the tobacco control landscape and changing social norms related to tobacco use since then, more recent estimates of favorable attitudes toward tobacco-free school policies could be different.

The findings from this study show that most US adults have a favorable attitude toward tobacco-free school grounds, including nearly 8 in 10 current tobacco users. However, differences in favorable attitudes toward such policies exist across states and subpopulation groups. Efforts to educate the public and policy makers at the state and local level about the dangers of tobacco use and the importance of tobacco-free school campus policies could be beneficial as part of a comprehensive approach to reduce youth tobacco use and preventing initiation.

## References

[1] US Department of Health and Human Services. The health consequences of smoking — 50 years of progress: a report of the Surgeon General. Atlanta (GA): Centers for Disease Control and Prevention, National Center for Chronic Disease Prevention and Health Promotion, Office on Smoking and Health; 2014.

[2] US Department of Health and Human Services. Preventing tobacco use among youth and young adults. Atlanta (GA): Centers for Disease Control and Prevention; 2012.

[3] Agaku IT , King BA , Husten CG , Bunnell R , Ambrose BK , Hu SS , ; Centers for Disease Control and Prevention (CDC). Tobacco product use among adults — United States, 2012–2013. MMWR Morb Mortal Wkly Rep 2014;63(25):542–7. 24964880 PMC5779380

[4] Arrazola RA , Singh T , Corey CG , Husten CG , Neff LJ , Apelberg BJ , ; Centers for Disease Control and Prevention (CDC). Tobacco use among middle and high school students — United States, 2011–2014. MMWR Morb Mortal Wkly Rep 2015;64(14):381–5. 25879896 PMC5779546

[5] US Department of Health and Human Services. Preventing tobacco use among young people: a report of the Surgeon General. Atlanta (GA): Centers for Disease Control and Prevention; 1994.

[6] Everett Jones S , Barrios LC , Hertz MF , Hall-Jordan LH . Safe and healthy school environment. In: Centers for Disease Control and Prevention. Results from the School Health Policies and Practices Study — 2012. Atlanta (GA): US Department of Health and Human Services, Centers for Disease Control and Prevention; 2013. p. 91–109. http://www.cdc.gov/healthyyouth/shpps/2012/pdf/shpps-results_2012.pdf.

[7] Barnett TA , Gauvin L , Lambert M , O’Loughlin J , Paradis G , McGrath JJ . The influence of school smoking policies on student tobacco use. Arch Pediatr Adolesc Med 2007;161(9):842–8. 10.1001/archpedi.161.9.842 17768283 PMC5758338

[8] West P , Sweeting H , Leyland A . School effects of pupils’ health behaviors: evidence in support of the health promoting school. Research Papers in Education 2004;19(3):261–91. 10.1080/02671522.2004.10058645

[9] Henderson M , Ecob R , Wight D , Abraham C . What explains between-school differences in rates of smoking? BMC Public Health 2008;8(1):218. 10.1186/1471-2458-8-218 18570635 PMC2442834

[10] Wray RJ , Jupka K , Berman S , Zellin S , Vijaykumar S . Young adults’ perceptions about established and emerging tobacco products: results from eight focus groups. Nicotine Tob Res 2012;14(2):184–90. 10.1093/ntr/ntr168 22110049

[11] Kaufman P , Griffin K , Cohen J , Perkins N , Ferrence R . Smoking in urban outdoor public places: Behaviour, experiences, and implications for public health. Health Place 2010;16(5):961–8. 10.1016/j.healthplace.2010.05.012 20576460

[12] Trinidad DR , Gilpin EA , Pierce JP . Compliance and support for smoke-free school policies. Health Educ Res 2005;20(4):466–75. 10.1093/her/cyg143 15572436

[13] US Department of Health and Human Services. The health consequences of involuntary exposure to tobacco smoke: a report of the Surgeon General. Atlanta (GA): Centers for Disease Control and Prevention, National Center for Chronic Disease Prevention and Health Promotion, Office on Smoking and Health; 2006. 20669524

[14] Azagba S , Kennedy RD , Baskerville NB . Smoke-free school policy and exposure to second-hand smoke: a quasi-experimental analysis. Nicotine Tob Res 2015. 25847291 10.1093/ntr/ntv077 PMC4710206

[15] Centers for Disease Control and Prevention, Office on Smoking and Health and ICF. 2009–2010 National Adult Tobacco Survey methodology report; 2011. http://www.cdc.gov/tobacco/data_statistics/surveys/nats/pdfs/methodology-report.pdf. Accessed December 3, 2015.

[16] Council of American Survey and Research Organizations. Code of standards and ethics for survey research, 2009. http://www.scribd.com/doc/17276719/code-of-standards-and-ethics for-survey-research-CASRO.

[17] Zhang X , Cowling DW , Tang H . The impact of social norm change strategies on smokers’ quitting behaviours. Tob Control 2010;19(Suppl 1):i51–5. 10.1136/tc.2008.029447 20382651 PMC2976550

[18] Procter-Scherdtel A , Collins D . Social norms and smoking bans on campus: interactions in the Canadian university context. Health Educ Res 2013;28(1):101–12. 10.1093/her/cys075 22722782

[19] US Department of Health and Human Services. Results from the School Health Policies and Practices Study — 2012. Atlanta (GA): US Department of Health and Human Services, Centers for Disease Control and Prevention, National Center for HIV/AIDS, Viral Hepatitis, STD, and TB Prevention, Division of Adolescent and School Health; 2013.

[20] International Agency for Research on Cancer. Evaluating the effectiveness of smoke-free policies. Lyon (FR): International Agency for Research on Cancer; 2009.

[21] US Department of Health and Human Services. Healthy people 2020: topics and objectives. Washington (DC): US Department of Health and Human Services; 2014. http://www.healthypeople.gov/2020/leading-health-indicators/2020-lhi-topics/Tobacco. Accessed December 2, 2015.

[22] Centers for Disease Control and Prevention. Guidelines for school health programs to prevent tobacco use and addiction. MMWR Recomm Rep 1994;43(RR-2):1–18. 8145711

[23] State Tobacco Activities Tracking and Evaluation (STATE) System. Atlanta (GA): Centers for Disease Control and Prevention and Health Promotion, Office on Smoking and Health; 2015. http://www.cdc.gov/statesystem. Accessed November 9, 2015.

[24] Educate America Act of 1994, 20 USCS §6081 (2001).

[25] Centers for Disease Control and Prevention. Best practices for comprehensive tobacco control programs — 2014. Atlanta (GA): US Department of Health and Human Services, Centers for Disease Control and Prevention, National Center for Chronic Disease Prevention and Health Promotion, Office on Smoking and Health; 2014.

[26] Institute of Medicine. Committee on reducing tobacco use: strategies, barriers, and consequences. In: Bonnie RJ, Stratton K, Wallace RB, editors. Ending the tobacco problem: a blueprint for the nation. Washington (DC): National Academies Press; 2007.

[27] Demissie Z , Brener ND , McManus T , Shanklin SL , Hawkins J , Kann L . School health profiles 2012: characteristics of health programs among secondary schools. Atlanta (GA): Centers for Disease Control and Prevention; 2013.

[28] Centers for Disease Control and Prevention (CDC). State-specific secondhand smoke exposure and current cigarette smoking among adults — United States, 2008. MMWR Morb Mortal Wkly Rep 2009;58(44):1232–5. 19910910

[29] Brown A , Moodie C , Hastings G . A longitudinal study of policy effect (smoke-free legislation) on smoking norms: ITC Scotland/United Kingdom. Nicotine Tob Res 2009;11(8):924–32. 10.1093/ntr/ntp087 19541947 PMC2711981

[30] Centers for Disease Control and Prevention (CDC). Vital signs: nonsmokers’ exposure to secondhand smoke — United States, 1999–2008. MMWR Morb Mortal Wkly Rep 2010;59(35):1141–6. 20829748

